# The Arrhythmogenic Spectrum of Mitral Valve Disease: Pathophysiology, Risk Stratification, and Surgical Management

**DOI:** 10.3390/jcm15020865

**Published:** 2026-01-21

**Authors:** Mariagrazia Piscione, Barbara Pala, Francesco Cribari, Walter Vignaroli, Jad Mroue, Vivek Mehta, Fadi Matar, Marco Alfonso Perrone

**Affiliations:** 1 Fondazione Policlinico Campus Bio-Medico, University of Rome, Via Alvaro del Portillo, 200, 00128 Rome, Italy; 2 PhD School of Applied Medical-Surgical Sciences, Tor Vergata University of Rome, Via Montpellier, 1, 00133 Rome, Italy; 3 UOC Cardiologia Ospedale, Istituto Dermopatico dell’Immacolata (IDI-IRCCS), 00167 Rome, Italy; 4 Department of Cardiac Surgery, GVM, San Carlo di Nancy, Via Aurelia, 275, 00165 Roma, Italy; 5 Division of Cardiology, University of South Florida, Tampa, FL 33620, USAfmatar@usf.edu (F.M.); 6 Division of Cardiology, Tor Vergata University of Rome, Via Montpellier, 1, 00133 Rome, Italy

**Keywords:** mitral valve prolapse, mitral annular disjunction, arrhythmic mitral valve prolapse, multimodality imaging, surgical treatment

## Abstract

Mitral valve prolapse (MVP) is generally associated with excellent long-term outcomes when MR is absent or mild. Nonetheless, a small proportion of patients exhibit a distinct arrhythmogenic susceptibility, characterized by complex ventricular ectopy, sustained ventricular arrhythmias (VAs), and in rare instances, sudden cardiac death (SCD). This subgroup—collectively referred to as arrhythmic MVP (AMVP)—has prompted renewed attention in identifying individuals at elevated risk. Among the structural alterations associated with MVP, mitral annular disjunction (MAD) has gained recognition as a major contributor to arrhythmic vulnerability, arising from the pathological separation of the posterior annulus from the adjacent ventricular muscle. Advances in multimodality imaging, including trans-thoracic echocardiography (TTE), cardiac magnetic resonance (CMR), and cardiac computed tomography (cCT), have significantly improved delineation of MAD and clarified its relationship to the broader MVP spectrum. Current evidence suggests that MVP, MAD, and AMVP should not be regarded as isolated conditions but as intersecting phenotypes within a shared pathological framework. In certain patients, especially those without established myocardial fibrosis, abnormal annular dynamics appear to constitute the primary arrhythmogenic driver and may diminish after surgical intervention. In others, persistent arrhythmias despite optimal repair reflect a fibrosis-based substrate. This review synthesizes contemporary insights into the anatomical, biomechanical, and electrophysiological interplay linking MVP, MAD, and ventricular arrhythmogenesis, emphasizing implications for imaging-based risk assessment and individualized surgical management strategies.

## 1. Introduction

Degenerative mitral valve disease includes a wide spectrum of structural abnormalities of the mitral valve (MV) apparatus, ranging from mild leaflet redundancy to advanced forms characterized by severe mitral regurgitation (MR) and left ventricular (LV) remodelling. Within this heterogeneous group of disorders, mitral valve prolapse (MVP) represents the most common phenotype, affecting approximately 2–3% of the general population [1]. In most individuals, and particularly in the absence of relevant MR, MVP is considered a benign condition with a favourable long-term prognosis [2]. Nonetheless, recent evidence indicates that this is not universally true: a minority of patients exhibit a distinct susceptibility to malignant ventricular arrhythmias (VAs) and, occasionally, sudden cardiac death (SCD), defining what has been termed the arrhythmic mitral valve prolapse (AMVP) phenotype [3]. Notably, the observation that MVP is overrepresented in autopsy series of young individuals who suffered SCD suggests the presence of specific structural or electrophysiological substrates predisposing to a malignant variant of the disease [4]. However, because of the low incidence of fatal events and the scarcity of large, prospective cohorts, the precise mechanisms and predictors of risk in MVP remain incompletely defined [4,5].

One structural abnormality that has emerged as a potential contributor to both degenerative and arrhythmogenic phenotypes is mitral annular disjunction (MAD) [6]. MAD corresponds to a systolic abnormal separation between the atrial wall–leaflet junction and the basal LV myocardium, often accompanied by a paradoxical systolic motion of the posterior annulus, described as “curling” [7,8]. Although once considered a rare pathological finding, MAD is now increasingly recognized through multimodality imaging, including transthoracic echocardiography (TTE), cardiac magnetic resonance (CMR), and cardiac computed tomography (cCT), all supported by pathological validation [9]. This integrative imaging approach has substantially advanced our understanding of the MVP spectrum and of the mechanisms contributing to arrhythmogenic remodelling. Importantly, MVP, MAD, and AMVP do not represent isolated entities but rather overlapping phenotypes within a continuum of MV disease [9]. Not all patients with MVP develop significant MR; not all develop arrhythmias; and the mechanisms predisposing to one or the other manifestation may diverge, partially overlap, or coexist within the same individual [9]. Understanding these interrelationships is crucial for accurate diagnosis, risk stratification, and therapeutic decision-making [9]. Importantly, emerging data suggest that MAD should not be viewed as a purely binary finding. Instead, its anatomical expression may be heterogeneous and dynamically dependent on imaging plane and timing, which has implications for prevalence estimates and for the interpretation of ‘MAD-positive’ MVP phenotypes.

In some patients, particularly in the absence of CMR-detected fibrosis, the abnormal annular dynamics associated with MAD act as the dominant trigger for VAs [10]. In these cases, surgical correction of the disjunction and restoration of normal annular–ventricular coupling may substantially reduce—and in selected cases suppress—the arrhythmic burden, supporting a mechanotransduction-driven and potentially reversible substrate [10]. Conversely, when VAs persist despite technically successful MV repair, advanced imaging frequently reveals areas of replacement fibrosis in the inferolateral wall or papillary muscles (PMs), indicating that the arrhythmogenic substrate has progressed to a fixed, scar-based phenotype no longer dependent on annular mechanics [10].

This review synthesizes current evidence on the anatomical and electrophysiological interplay between MVP, MAD, and the AMVP phenotype, emphasizing how multimodality imaging and surgical response patterns can clarify arrhythmogenic mechanisms.

We further discuss how identifying MAD—and assessing its relationship with myocardial fibrosis—should inform modern clinical and surgical decision-making.

## 2. Literature Search Strategy and Article Selection

A comprehensive literature search was performed in PubMed to identify relevant studies published between January 2017 and January 2025 focusing on MVP, MAD, and their arrhythmogenic implications. The search strategy included the following terms, used alone or in combination: “mitral valve prolapse and mitral annular disjunction”, “mitral annular disjunction and surgery”, “mitral annular disjunction and arrhythmias”, and “mitral annular disjunction and arrhythmias and ablation”. This search yielded a total of 544 records, all of which were screened by title and abstract.

After initial screening, 400 records were excluded because of limited relevance to the aims of the review, absence of clinically or biologically meaningful outcomes, or redundancy with more comprehensive and up-to-date review articles. The full texts of the remaining 144 articles were assessed for eligibility. Of these, 77 articles were excluded for the following reasons: purely descriptive case reports, articles not written in English, inability to distinguish association from causation, or limited clinical relevance.

**Figure 1:**
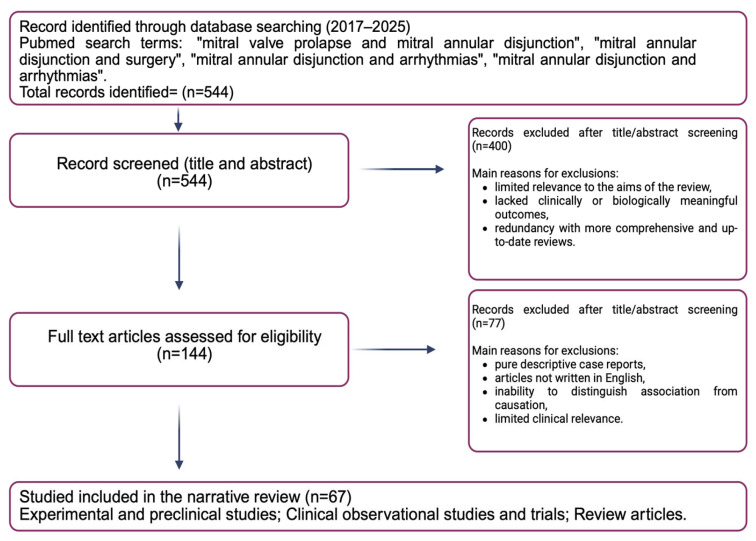
Literature review (Created in BioRender.com).

Overall, 67 studies met the predefined inclusion criteria and were included in the narrative synthesis. In addition, 19 further relevant studies were identified through manual screening of reference lists and contextual relevance to key imaging or clinical aspects of the AMVP spectrum. Therefore, a total of 86 studies were included in the present narrative review, comprising experimental and preclinical investigations, clinical observational studies and trials, and review articles [Figure 1].

## 3. Mitral Valve Prolapse

### 3.1. Definition and Etiologic Subtypes

MVP is defined as a systolic displacement of one or both mitral leaflets ≥ 2 mm above the plane of the mitral annulus in the parasternal long-axis view [8,10].

**Figure 2:**
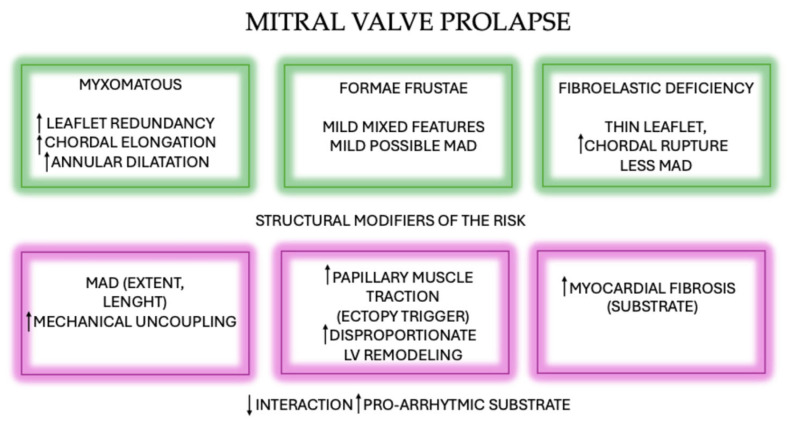
Conceptual framework of mitral valve prolapse phenotypes and arrhythmogenic risk. Mitral valve prolapse encompasses a degenerative continuum ranging from fibroelastic deficiency to advanced myxomatous disease, with intermediate (“formae frustae”) phenotypes. Structural modifiers—including mitral annular disjunction (MAD), papillary muscle traction, and left ventricular remodelling—modulate arrhythmic susceptibility. MAD acts as a mechanical driver that amplifies systolic stress and ventricular uncoupling, whereas myocardial fibrosis represents a fixed arrhythmogenic substrate. The interaction between valvular morphology, annular–ventricular mechanics, and myocardial remodelling ultimately determines the development of a pro-arrhythmic phenotype. Abbreviations: ↑ increase, ↓ reduce, LV: left ventricle, MAD: mitral annular disjunction.

Two main etiologic patterns are recognized. Myxomatous MVP (Barlow’s disease) is characterized by redundant leaflet tissue, thickened and elongated chordae, annular dilatation, and often mild calcification, typically with a low risk of chordal rupture, whereas fibroelastic deficiency, defined by focal or diffuse thinning of the leaflets and chordae, with elongation or rupture leading to varying degrees of MR [11]. Between these two entities lies a broad spectrum of intermediate or “formae frustae” phenotypes, exhibiting overlapping morphological features and representing transitional expressions of degenerative MV disease continuum [11,12] [Figure 2].

### 3.2. Epidemiology

MVP is the most common valvular abnormality in Western populations, with a prevalence ranging from 0.6% to 3.1%, depending on the diagnostic criteria and population studied [12]. Its frequency increases with age, shows a slight female predominance, and is comparable across ethnic groups [13]. The prevalence of MVP among competitive athletes is comparable to that observed in the general population [13]. Although most MVP cases are sporadic, familial aggregation has been consistently reported [13]. Having an affected parent increases the risk of MVP approximately five-fold, and first-degree relatives show a 30–50% likelihood of being affected [12,13]. A subset of MVP occurs in the context of heritable connective tissue disorders, reflecting abnormal synthesis and organization of collagen and extracellular matrix proteins. Myxomatous MVP is reported in up to 75% of patients with Marfan syndrome aged > 60 years, approximately 45% of those with Loeys–Dietz syndrome, and around 6% of patients with Ehlers–Danlos syndrome [13].

### 3.3. Clinical Outcomes and Sudden Cardiac Death

As already stated, the clinical course of MVP is predominantly determined by the severity of MR and its effects on LV size and function [3]. In the general MVP population, the annual risk of SCD is low, ranging between 0.2% and 0.4%; however, it increases to approximately 1.8% per year in patients with severe MR due to a flail leaflet and rises further to 7–8% per year among those with advanced heart failure (functional NYHA class III–IV) [14]. Although the absolute risk remains modest, these figures highlight the presence of a distinct malignant MVP phenotype, in which arrhythmogenic remodelling—rather than MR severity alone—appears to drive adverse outcomes [14,15,16,17]. Supporting this concept, recent large cohorts of patients with isolated MVP systematically evaluated with 24 h Holter monitoring, TTE, and clinical follow-up have shown that VAs are common but generally mild [18]. Nevertheless, when VAs are severe, they are independently associated with increased long-term mortality, regardless of the degree of MR [18]. Taken together, these observations suggest that a subset of MVP patients progressively develops a pro-arrhythmic structural and electrical substrate, which may remain clinically silent for years but eventually manifests with sustained or complex Vas [18]. These findings indicate that arrhythmic risk in MVP is shaped by several structural and myocardial factors, with MAD initially emerging as a leading candidate in explaining the malignant phenotype [19]. In recent years, considerable attention focused on MAD as a potential key driver of arrhythmogenesis; however, as more robust imaging and new data have become available, its role has been progressively reconsidered [19]. The next section places this evolving perspective into context, outlining how interest in MAD has shifted from early enthusiasm to a more nuanced understanding of its contribution within a broader and multifactorial arrhythmogenic substrate.

## 4. Mitral Annular Disjunction

### 4.1. Definition

**Figure 3:**
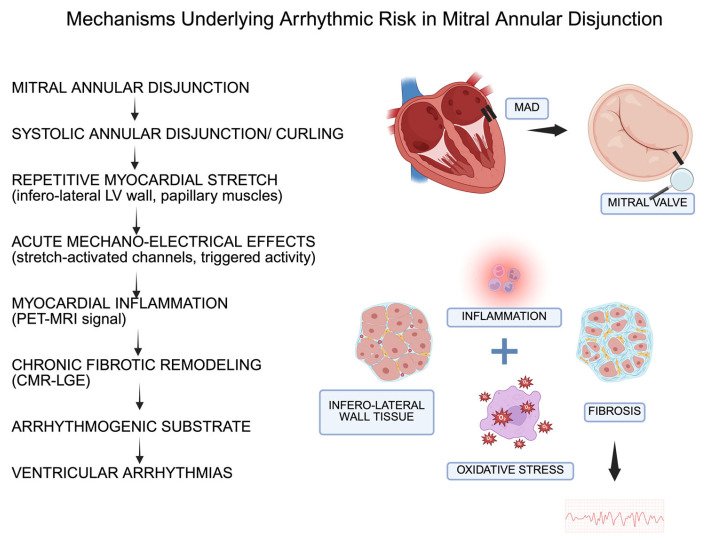
Pathophysiological continuum linking mitral annular disjunction to ventricular arrhythmias (Created in BioRender.com). Abbreviations: CMR: Cardiac Magnetic Resonance, LGE: Late Gadolinium Enhancement, LV: left ventricle, PET-MRI: Positron Emission Tomography-Magnetic Resonance Imaging.

MAD is defined as a systolic separation between the ventricular myocardium and the hinge point of the posterior mitral leaflet [16,17] [Figure 3]. As a matter of fact, the presence of MAD results in a loss of mechanical continuity between the annulus and ventricular myocardium while electrical continuity is maintained [13,15,16]. The circumferential extension of MAD is anteriorly limited by the mitro-aortic fibrous continuity; therefore, it is typically observed at the posterior leaflet insertion [14,16,17,18]. The disjunction can extend laterally beneath one or more posterior scallops, most frequently under the P2 scallop [16]. Notably, despite its growing recognition, there is currently no universally accepted quantitative threshold defining pathological MAD [16]. As a result, MAD should be regarded as a morphological continuum rather than a binary anatomical abnormality [17].

### 4.2. Pathogenesis and Diagnosis of MAD

After delineating the anatomical and functional features of MAD, a key question concerns its prevalence and the reliability of its identification in clinical practice—two issues that remain the focus of ongoing debate [19,20].

When evaluated with advanced imaging modalities, MAD has frequently been described as a common anatomical variant of the posterior mitral annulus in healthy individuals even observed in individuals without MVP. Indeed, some studies have found no association between MAD and SCD in patients with MVP during long term follow-up [20,21,22,23]. In addition, reported prevalence of MAD in MVP varies extraordinarily widely, from 76% to 96% in selected datasets [21,22]. This heterogeneity reflects several methodological limitations.

First, definitions of MAD differ substantially across centres, making comparison between studies difficult [23]. Prevalence estimates may vary by as much as 300% depending on the imaging technique and diagnostic cut-offs employed [24]. Multiplanar and high-resolution modalities identify MAD far more frequently than standard TTE [24]. Accordingly, careful frame-by-frame assessment and multiplanar/3D imaging can help distinguish true MAD from pseudo-MAD related to redundant leaflet tissue or plane dependency, improving diagnostic reproducibility across centres [9].

Second, the absence of an agreed-upon threshold for MAD extent complicates attempts to establish its true epidemiology [24]. Reliable identification requires a zoomed long-axis view with high frame rate, enabling frame-by-frame analysis of annular motion [25]. The distance between the posterior leaflet hinge point and the upper LV myocardium is measured at end-systole, and dynamic imaging is essential to avoid confusing redundant leaflet tissue with true disjunction [26]. Although formal guidelines are currently lacking, recent European Heart Rhythm Association (EHRA) documents emphasize the importance of a standardized imaging approach for MAD assessment suggesting sagittal imaging planes—particularly parasternal long-axis views [27]. Moreover, according to this 1 expert consensus, TTE remains the preferred first-line imaging modality for MAD evaluation due to its wide availability and feasibility in routine clinical practice, whereas CMR is reserved for patients with suboptimal echocardiographic windows or inadequate image quality [27]. The circumferential and width extent of MAD should be further evaluated in apical views, and serial two-dimensional TTE over time may be useful to explore MAD progression and its potential relationship with adverse structural and arrhythmic remodelling [27]. Historically, the association between MAD and MVP originates from early pathological and TTE descriptions that suggested a close relationship between the two conditions [20]. Yet the earliest studies linking MAD to life-threatening VAs were limited by cross-sectional designs and strong selection bias, often focusing on patients with an already high arrhythmic burden [20]. More recent longitudinal analyses have challenged the prognostic value of MAD when assessed in standard long-axis views [21]. In one echo-based cohort, MAD did not increase mortality over a 10-year follow-up, although it remained associated with a composite arrhythmic endpoint [21]. Importantly, this association persisted even after MV surgery and apparent MAD resolution, suggesting that MAD may sometimes act as a marker rather than a direct arrhythmogenic driver [21]. Likewise, in a CMR-based cohort, MAD did not predict arrhythmic events, whereas myocardial fibrosis emerged as the strongest determinant of arrhythmogenic risk [23]. That is the reason why in the absence of standardized diagnostic criteria, the mere identification of MAD should not be interpreted as an automatic marker of arrhythmic risk [23]. Rather, “MAD-positive” findings need to be contextualized within a comprehensive phenotypic assessment that includes leaflet morphology, ventricular remodelling, myocardial fibrosis, and arrhythmic burden [23]. Overdiagnosis of MAD—particularly when based on single-plane or non-dynamic imaging—may lead to unnecessary alarm and inconsistent risk stratification across centres [24]. These limitations underscore the need for prospective, multicenter studies adopting unified imaging protocols, predefined measurement timing within the cardiac cycle, and reproducible cut-off values for pathological disjunction. Such an approach is essential to clarify the true prevalence of clinically relevant MAD and to define its independent prognostic role in AMVP [24]. Importantly, this need for diagnostic caution does not negate the potential pathophysiological relevance of MAD [24]. Even so, extensive MAD may still identify regions susceptible to arrhythmogenic remodelling.

From a mechanistic standpoint, a large longitudinal extent of MAD results in marked systolic “outpouching” and apical displacement of the atrial–leaflet junction, accompanied by exaggerated systolic motion—or curling—of the proximal inferolateral LV wall [24]. The abnormal mechanical forces transmitted to the ventricular myocardium and PMs can induce electrophysiological disturbances, including shortened action potential duration and stretch-mediated early afterdepolarizations [25]. Over time, such mechanical–electrical interactions may activate molecular pathways that promote myocardial fibrosis [15]. In turn, fibrosis then provides a structural substrate for re-entrant VAs and has consistently been associated with malignant VAs and adverse outcomes in degenerative MV disease [25,26,27]. Supporting this hypothesis, several studies have demonstrated a strong correlation between the longitudinal extent of MAD and fibrosis burden on CMR [10], and MAD lengths >8.5 mm have been linked to VAs in select cohorts [25] [Figure 2].

### 4.3. Epidemiology

**Figure 4:**
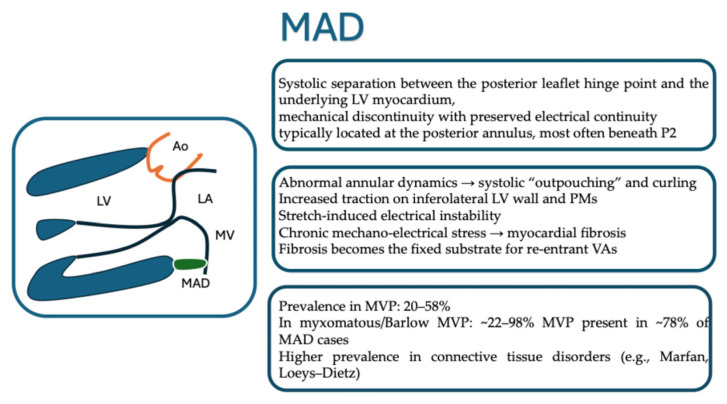
Key anatomical and pathophysiological features of mitral annular disjunction. Abbreviations: MAD: mitral annular disjunction, MVP: mitral valve prolapse, PMs: papillary muscles. LV: left ventricle, VAs: ventricular arrhythmias.

Within this context of methodological heterogeneity, reported epidemiological data on MAD warrant careful interpretation [Figure 4]. Among patients with MVP, MAD has been reported in 20–58% of cases, with a pooled prevalence of 32.6% across three studies [28,29,30]. In the subgroup with myxomatous or Barlow-type MVP, prevalence estimates are even higher, ranging from 21.8% to 98% (pooled prevalence 50.8% in three studies) [31]. Conversely, among individuals with MAD, the presence of MVP has been documented in approximately 78%. Syndromic conditions also show a substantial burden of MAD, with reported rates of in Marfan syndrome and 40% in patients carrying Loeys–Dietz mutations [32]. In these contexts, MAD often reflects a more severe disease phenotype, characterized by increased arrhythmic events, a higher likelihood of requiring MV intervention, and—as noted particularly in extensive MAD—an elevated arrhythmic burden [31,32] [Table 1].

## 5. The Arrhythmic Mitral Valve Phenotype

Having examined the structural abnormalities of MVP and the modulators of arrhythmogenic risk—particularly the extent of MAD and the degree of myocardial fibrosis—the next step is to frame how these elements converge clinically [32]. Not all patients with MVP and MAD develop malignant VAs; rather, only a subset displays a constellation of electrocardiographic, imaging, and symptom-based markers that define what is now recognized as the AMVP phenotype [32].

AMVP represents a clinically meaningful entity in which mechanical abnormalities of the MV apparatus, dynamic annular–ventricular interactions, and varying degrees of myocardial involvement create a substrate capable of sustaining complex or life-threatening VAs [28,29]. Importantly, AMVP is not defined by the presence of MAD alone, nor by MR severity, but by the interaction of these features with electrical instability, myocardial remodelling, and characteristic phenotypic markers [29]. Accordingly, two main phenotypes can be distinguished: the AMVP related to severe degenerative MR and the AMVP related to myxomatous disease, independent of MR severity [29].

### 5.1. AMVP Related to Severe Degenerative Mitral Regurgitation

Patients with MVP and severe MR exhibit a significantly increased risk of overall mortality, including a higher incidence of SCD, compared with the general population, irrespective of leaflet morphology [30]. Although atrial arrhythmias, reduced LV systolic function, and advanced heart failure symptoms amplify this risk, excess mortality and SCD remain evident even in the absence of these additional markers and with only moderate-to-severe MR [30]. Surgical correction of degenerative MR has consistently been associated with a marked reduction in VAs and SCD, and with restoration of life expectancy through normalization of long-term mortality [31,32,33].

### 5.2. AMVP Related to Myxomatous Disease

Morphologically, this AMVP phenotype is characterized by advanced myxomatous degeneration, with pronounced leaflet redundancy and increased leaflet thickness and length [34]. MAD is frequently present and often extensive, and although bileaflet involvement is common, it is not a mandatory feature [34]. Importantly, MR may range from absent to severe, underscoring that regurgitation is not the primary determinant of arrhythmic risk in this setting [34]. This phenotype occurs across a broad age spectrum and is consistently associated with a higher frequency of VAs [35]. Notably, the arrhythmic profile of these patients appears to be independent of sex, MR severity, LV systolic function, and even the presence or absence of bileaflet prolapse, highlighting the uniqueness of this high-risk substrate [35]. Although patients with this phenotype but without overt arrhythmias remain at increased future risk, they do not need immediate intervention, instead, it is important to do repeated and prolonged rhythm surveillance [36]. Overall, even within this at-risk AMVP subset, the clinical presentation is highly heterogeneous, underscoring the importance of accurately identifying arrhythmic complications and tailoring management to the individual patient [36].

## 6. AMVP: Clinical and Imaging Profile

### 6.1. Symptoms

Syncope is relatively uncommon in unselected MVP populations, but it is reported far more frequently among patients with malignant VAs or SCD [37]. In large clinical cohorts, syncope is strongly associated with documented severe VAs; therefore, unexplained syncope—especially when recurrent—significantly heightens the suspicion for a malignant arrhythmic substrate [37]. Conversely, palpitations and chest pain are common but occur at similar rates in MVP patients with and without VAs and are therefore less discriminative [37].

### 6.2. Electrographic Abnormalities

Electrocardiographic abnormalities, particularly T-wave inversion in the inferior and lateral leads, represent an important marker of arrhythmic risk in MVP [38,39]. These repolarization changes are believed to reflect abnormal regional mechanics, including PMs stretch and localized disturbances in electrical recovery within the basal left ventricle [38,39]. However, the utility of T-wave inversion as a specific marker of high arrhythmic risk is debatable, given that it may be observed in up to 40% of patients with MVP, reducing its ability to distinguish truly high-risk individuals [40].

In addition, subjects with MVP have been reported to exhibit longer QT intervals than control populations in some, though not all, studies [40,41]. When present, QT prolongation correlates with more pronounced leaflet prolapse and increased anterior leaflet thickness [41]. Moreover, fragmented QRS, defined by the presence of additional R′ waves, notching of the R or S waves, or more than one R′ in two contiguous leads, has been recognized as a marker of localized myocardial scar and has been associated with increased arrhythmic mortality and SCD across multiple populations [42].

The detection of premature ventricular contractions (PVCs) on serial standard ECGs generally reflects a substantial burden of ventricular ectopy, and the documentation of non-sustained ventricular tachycardia (VT) is considered a higher-risk marker. In MVP, PVC morphologies often suggest origins from the PMs, the mitral annulus, or the basal LV segments, although fascicular patterns can also be observed. Ectopy arising from the outflow tracts is likewise common [43].

### 6.3. Echocardiographic Assessment

Echocardiography remains the cornerstone for structural assessment [44]. Morphologic features associated with an AMVP phenotype include severe myxomatous degeneration with redundant, thickened leaflets, multi-segment bileaflet prolapse, and the presence of MAD [44]. Importantly, bileaflet prolapse alone—though common—does not independently predict SCD in large cohorts [44]. Whether MAD length predicts more frequent arrhythmias is unclear as are MAD-associated arrhythmia mechanisms, although myocardial fibrosis is often noted with longer MAD and hypothesized as causative [45].

### 6.4. Cardiac MRI Evaluation

Cardiac MRI adds substantial value to risk stratification owing to its ability to detect focal fibrosis through late gadolinium enhancement (LGE) [46]. Fibrosis is relatively common in MVP and typically involves myocardial regions adjacent to the mitral apparatus—such as the basal inferolateral wall, inferior wall, and PMs—and has been consistently linked to complex VAs [47,48]. MRI-derived indicators of diffuse interstitial remodelling, including extracellular volume expansion and prolonged post-contrast T1 times, are also associated with arrhythmic risk and may represent earlier stages of disease that precede the emergence of replacement fibrosis on LGE [49]. Recent hybrid positron emission tomography-magnetic resonance imaging (PET–MRI) studies further expand this concept by demonstrating that many patients with MVP and only mild-to-moderate MR exhibit focal myocardial inflammation that frequently overlaps with regions of fibrosis [50].These findings suggest that inflammatory activity may contribute to the evolution of the arrhythmogenic substrate and could help explain why malignant VAs and SCD often occur in patients without severe MR [51]. The coexistence of inflammation and scar highlights a dynamic pathological process affecting the mitral apparatus and surrounding myocardium, reinforcing the need for multimodality imaging to capture the full extent of myocardial involvement [52].

### 6.5. Cardiac CT

cCT can also aid in detecting MAD and delineating its extension—particularly toward the P1 and P3 scallops—thanks to its high spatial resolution. Although temporal resolution is inferior to TTE, cCT provides sub-millimeter three-dimensional datasets that are highly informative for characterizing the fibrous components of the MV apparatus and identifying mitral annular calcification, information that may be particularly relevant when planning MV repair [27,53].

## 7. Risk Stratification

Arrhythmia screening is central to risk assessment in patients with MVP and MAD, as the degree of arrhythmia documented on ambulatory monitoring ultimately determines both the intensity of follow-up and the need for therapeutic action [54]. Importantly, the presence of MAD does not uniformly confer an arrhythmic phenotype, nor do all detected VAs translate into excess mortality [55]. Therefore, MVP in the absence of significant degenerative MR, clinical complications, or high-grade VAs should prompt reassurance rather than warning [56]. Conversely, the identification of phenotypic markers associated with increased arrhythmic susceptibility places the patient into a higher-risk category. In this setting, even mild-to-moderate PVCs may warrant more frequent or prolonged monitoring [57,58,59,60,61]. Crucially, it is the arrhythmia itself, rather than the MVP morphology alone, that dictates clinical decision-making, and intervention is justified only in the presence of severe VAs—particularly when accompanied by syncope or pre-syncope [62].

Clinical presentations such as unexplained syncope or pre-syncope should heighten suspicion for significant VAs [63]. Imaging or electrocardiographic markers—including T-wave inversion, pronounced myxomatous degeneration, redundant leaflets, presence of MAD, or LGE on CMR—further strengthen this concern [63]. Sustained VT, spontaneous polymorphic non sustained VT (NSVT), or rapid monomorphic NSVT (>180 bpm) represent high-risk patterns and are considered warning signs for SCD. In such contexts, intensified rhythm surveillance is appropriate, ideally through long-term Holter devices or implantable loop recorders (ILRs), which maximize diagnostic yield [62]. The overall risk–benefit profile of this intensified strategy remains incompletely defined.

In contrast, asymptomatic patients with no complex VAs on initial Holter require only periodic surveillance, typically managed in primary care [62].

**Figure 5:**
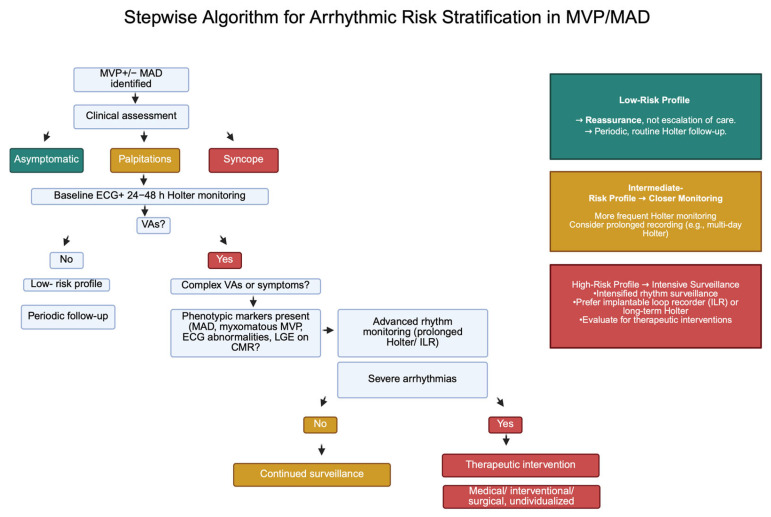
Proposed stepwise algorithm for arrhythmic risk stratification in MVP/MAD (Created in BioRender.com). Abbreviations: CMR: Cardiac Magnetic Resonance, ECG: electrocardiogram, ILR: implantable loop recorder, LGE: late gadolinium enhancement, MAD: mitral annular disjunction, MVP: mitral valve prolapse, VAs: ventricular arrhythmias.

Ultimately, the severity of ventricular arrhythmias detected on Holter remains the principal determinant of both the intensity of subsequent rhythm monitoring and the threshold for medical or surgical intervention [Figure 5].

## 8. AMVP Management

The first consensus document on the management of AMVP, despite persisting gaps in knowledge, provides clarification regarding diagnostic tools and therapeutic goals [62]. Long-term follow-up and medical therapy are advocated for the frequent low-risk VAs observed in MVP, but although a high burden of ventricular ectopy is associated with increased mortality in the general population, there is currently no evidence that prophylactic suppression of ventricular extra-systole in asymptomatic MVP patients is beneficial [63]. Medical therapy aimed at suppressing PVCs is recommended in cases of suspected PVC-induced cardiomyopathy, reduced LV ejection fraction unrelated to degenerative MR, and in symptomatic patients irrespective of LV function [64]. Beta-blockers and verapamil improve symptoms but achieve only modest reductions in PVC burden. Flecainide, propafenone, and amiodarone provide more potent suppression of ventricular ectopy and are frequently associated with improvement in LV function [65]. The potential benefits of amiodarone must be carefully weighed against its well-known long-term adverse effects [66]. Sotalol may reduce PVC burden but does not appear to consistently improve LV function [66]. Cases of PVC-induced ventricular fibrillation (VF) have been reported, with triggers originating from Purkinje fibres located within the PMs or fascicular system [67]. Some of these episodes have proven refractory to conventional antiarrhythmic therapy, including in patients with MVP [67]. Quinidine may be beneficial in treating short-coupled PVCs that precipitate polymorphic VT, owing to its modulatory effects on the Purkinje network. However, its efficacy has not been evaluated prospectively nor specifically in the MVP population [68].

### 8.1. Prevention of SCD with ICD

Established indications for primary and secondary prevention with implantable cardioverter-defibrillators (ICDs) apply equally to patients with MVP [68]. For primary prevention, guidelines recommend ICD implantation in individuals with symptomatic heart failure and an ejection fraction ≤ 35% despite three months of optimal medical therapy [68]. However, whether this criterion is frequently met in MVP or degenerative MR is unclear, as such degrees of systolic dysfunction are relatively uncommon in this population [68]. For secondary prevention, ICD implantation is indicated in patients with MVP who have survived sudden cardiac arrest or have experienced VF or sustained VT in the absence of reversible causes [68]. Primary prevention in AMVP is more nuanced, as no randomized trials have demonstrated a clear benefit in any MVP subgroup [68]. In the absence of definitive evidence, ICD implantation should be strongly considered in patients with unexplained syncope accompanied by high-risk ventricular arrhythmias identified on ECG, Holter monitoring, implantable loop recorder tracing, or possibly during exercise testing [68]. Given the lack of data specific to AMVP, the choice between subcutaneous and transvenous ICD systems should follow the same principles used for other arrhythmic conditions [53]. This decision requires careful assessment of the index arrhythmia, the anticipated efficacy of anti-tachycardia pacing, and any potential need for bradycardia pacing support [53]. As always, the risk of inappropriate shocks must be weighed against the risks associated with lead failure and endovascular infection [53,68].

### 8.2. Prevention of SCD with Transcatheter VT Ablation

Transcatheter VT ablation has been performed in patients with AMVP, most commonly targeting PMs or Purkinje system foci, and can be successful [53,69,70]. Nonetheless it has to be considered that catheter ablation of PMs foci remains technically demanding due to constant motion of the target structures and the difficulty in maintaining stable catheter–tissue contact. As a result, successful ablation often requires the use of intracardiac echocardiography for precise anatomical guidance, contact force–sensing catheters, and in some cases cryoablation to enhance catheter stability and improve energy delivery [71,72]. However, long-term success rates for ventricular extra-systole ablation in MVP range from 60% to 84%, and its role in preventing sudden arrhythmic death remains uncertain [53]. PVCs ablation has been employed in patients with refractory symptoms due to frequent extra-systole or with extra-systole-induced LV dysfunction, but the impact of these interventions on long-term outcomes is unclear. In patients with AMVP, frequent extra-systole, severe degenerative MR, and reduced LV function, guideline-based indications for surgical MV repair should not be delayed by considerations of arrhythmia ablation [53].

### 8.3. Mitral Valve Surgery

MV surgery represents the cornerstone therapy for patients with MVP and severe MR who are symptomatic or exhibit LV systolic dysfunction [73]. The primary objective of surgical intervention is the restoration of valve competence, prevention of adverse LV remodelling, and improvement of long-term survival [73]. As understanding of the AMVP phenotype has expanded, attention has increasingly shifted toward the potential role of MV surgery in modifying the ventricular arrhythmia burden, particularly in patients with MAD or marked systolic traction on the PMs [74].

#### 8.3.1. Surgical Restoration of Annular–Ventricular Coupling and Its Arrhythmic Implications

A substantial proportion of VAs in MVP appear to originate from abnormal mechanical forces exerted on the MV apparatus—mechano-transduction phenomena involving exaggerated leaflet motion, annular hypermobility, systolic curling of the posterior annulus, and traction on the PMs [75]. In this context, MAD represents a structural substrate that amplifies these forces and may contribute to abnormal annular configuration and coaptation loss in myxomatous MVP, providing a rationale for surgical strategies aimed at restoring annular–ventricular coupling when clinically indicated [75]. Surgical annuloplasty re-establishes the continuity between the annulus and the basal LV myocardium, mitigating the paradoxical systolic excursion and the excessive tension transmitted to the PMs [75].

Several retrospective series have reported a notable reduction in VAs burden following MV repair, supporting the concept that in patients whose arrhythmias are predominantly driven by abnormal annular dynamics, correction of the mechanical disturbance may reverse the arrhythmogenic process [76,77]. In the study by Essayagh et al., MV surgery rendered the risk of postoperative VT statistically non-significant in patients with MAD [21]. Similarly, Naksuk et al. demonstrated that surgical repair was associated with VAs reduction primarily in younger patients (<60 years), suggesting that earlier intervention—prior to the development of irreversible myocardial fibrosis—confers the greatest arrhythmic benefit [74]. This pattern supports the idea that, as MAD progresses, persistent mechanical overload may lead to the development of irreversible myocardial fibrosis—an arrhythmogenic substrate that does not resolve with surgical correction [74] [Table 2].

#### 8.3.2. Identifying a Potential “Window of Opportunity” for Arrhythmia-Modifying Surgery

The concept of an earlier intervention to mitigate “MAD-driven” arrhythmias should be framed as a hypothesis rather than a guideline-endorsed indication, given the paucity of prospective data and the absence of standardized thresholds [74]. Clinically, a potential “window of opportunity” may be suspected when a patient displays an active arrhythmic phenotype—recurrent complex ventricular ectopy, arrhythmic syncope/pre-syncope, or frequent symptomatic PVCs refractory to medical therapy—together with mechanical substrates/triggers typical of the AMVP spectrum (marked myxomatous degeneration, exaggerated annular dynamics/curling, PMs traction, and/or extensive MAD), in the absence of advanced scar burden [75].

In this setting, CMR is pivotal for surgical planning and risk contextualization. Detection of LGE (particularly involving the PMs or inferolateral LV) identifies an established arrhythmogenic substrate and may indicate that the disease process has progressed beyond a purely “trigger-dominant” stage; conversely, the absence of LGE does not exclude risk but may support the notion of an earlier, potentially modifiable phase characterized by mechanical stretch and electrical instability [47]. CMR further refines anatomy (bileaflet prolapse, annular geometry), evaluates LV remodelling, and can quantify fibrosis distribution, all of which may inform the likelihood that valve repair will reduce mechanical triggers [48].

Electrophysiological testing should be viewed as complementary rather than diagnostic of a specific surgical indication. EP study and/or electroanatomic mapping can help localize dominant ectopic foci, guide catheter ablation when appropriate, and support integrated decision-making within a multidisciplinary team [68]. At present, referral for surgery primarily to suppress arrhythmias in patients without severe MR should be restricted to selected cases—typically those with recurrent high-grade VAs or arrhythmic syncope despite optimized medical/ablative strategies, and with valve anatomy amenable to durable repair—while acknowledging that robust criteria defining this “window” require prospective multicenter validation [69].

#### 8.3.3. The Central Role of Myocardial Fibrosis in Determining Surgical Response

Recent multimodality imaging studies have reinforced the notion that postoperative arrhythmic outcomes are deeply conditioned by the underlying myocardial substrate. As noted, patients with established inferolateral or PMs fibrosis are less likely to experience meaningful suppression of VAs after mitral repair, reflecting the persistence of a fixed scar-based arrhythmogenic circuit that is no longer dependent on mechanical stretch [74]. This paradigm is further expanded by the findings of Miller et al., who demonstrated that even patients with degenerative MVP and only mild or moderate MR—traditionally considered at lower mechanical risk—frequently exhibit evidence of myocardial injury [78]. In their prospective hybrid PET/MRI study, focal or focal-on-diffuse FDG uptake, a surrogate for active myocardial inflammation, was detected in 83% of patients, and in three-quarters it colocalized with areas of LGE [78]. These observations lend support to the hypothesis that myocardial inflammation and microstructural injury may precede, accompany, or even potentiate the development of replacement fibrosis, thereby contributing to arrhythmogenesis independently of MR severity [78].

Taken together, the postoperative behaviour of VAs and the imaging findings from studies such as Miller’s suggest that the arrhythmic substrate in MVP may evolve through a continuum—from reversible, stretch-driven abnormalities to a fibrotic and inflammatory phenotype [78]. In patients at the early, predominantly mechanical end of this spectrum, surgical restoration of annular–ventricular coupling may sufficiently suppress arrhythmic triggers [78]. Conversely, when PET or CMR reveal established fibrosis or persistent inflammatory activity, surgery alone is unlikely to normalize arrhythmic risk. In this subgroup, postoperative management should incorporate tailored strategies including ICD implantation, extended arrhythmia surveillance, and, when appropriate, adjunctive electrophysiological interventions [75]. Ultimately, these insights underscore the pivotal role of preoperative imaging—not only to define anatomy and valve pathology but to determine whether the arrhythmogenic milieu is potentially reversible or already consolidated into a chronic substrate that requires multimodal, individualized therapy [75].

#### 8.3.4. Surgical Correction of MAD

From a technical standpoint, surgical management of MAD has progressively shifted toward approaches that preserve the native valvular and subvalvular apparatus rather than relying on extensive resection [76]. Historically, concerns were raised that the abnormal systolic expansion and flattening of the annulus, together with relative leaflet and chordal tissue deficiency, might compromise the durability of mitral repair or hinder secure implantation of an annuloplasty ring on a displaced annular plane [79,80]. Early corrective strategies therefore focused on re-anchoring the posterior annulus with sutures and resecting redundant tissue. More contemporary practice, however, aligns with the broader paradigm that maintaining structural integrity results in more physiological biomechanics; thus, respect-based repair using artificial chordae and ring annuloplasty has become the dominant technique, even in the presence of MAD [81]. Consistent with this evolution, available data—including the cohort analyzed by Lodin et al.—indicate that surgical repair reliably eliminates anatomical MAD without increasing the risk of recurrent disjunction or repair failure [79]. In this context, MAD appears surgically correctable using standard modern repair principles, and its presence does not in itself compromise the technical success or durability of mitral valve reconstruction, despite the persistent arrhythmic substrate that may remain postoperatively [78].

#### 8.3.5. The Limitation of Transcatheter Edge-to-Edge Repair

Transcatheter edge-to-edge repair (TEER) has demonstrated survival benefits in high-risk patients with severe degenerative MR [82]. However, TEER does not address the anatomical separation between annulus and myocardium, the defining feature of MAD. Since annular–ventricular uncoupling remains uncorrected, TEER cannot be expected to mitigate the mechanical triggers of arrhythmias [82]. Therefore, in patients with AMVP and significant MAD, surgical repair remains the gold standard approach.

#### 8.3.6. Intraoperative Cryoablation and Adjunctive Therapies

Some centres have incorporated intraoperative cryoablation targeting focal arrhythmogenic areas—typically the PMs or basal infero-lateral LV wall—during MV surgery [80]. Although encouraging as a complementary strategy, data remain limited to small series, and systematic evaluation is still required [80]. The potential integration of surgical repair with targeted arrhythmia modification may become particularly relevant in patients with focal triggers identifiable preoperatively [80].

#### 8.3.7. A More Nuanced Understanding of the Surgical Role in AMVP

The cumulative evidence suggests that MV surgery may serve a dual function in patients with MVP and arrhythmic risk: hemodynamic correction, alleviating MR and stabilizing LV, remodelling arrhythmogenic modulation, particularly when VAs arise from MAD-related mechanical forces [81].

However, the arrhythmic benefit depends critically on the timing of intervention and the underlying substrate. In early, dynamic disease, where MAD-driven mechanical stretch predominates, surgical repair may substantially reduce or abolish VAs. In advanced disease, where fibrosis is established, surgical intervention may improve symptoms and LV remodelling but does little to alter arrhythmic risk [82].

Consequently, a comprehensive, multimodality evaluation integrating clinical data, ECG, Holter monitoring, CMR is essential to guide individualized decision-making. The ability to distinguish a reversible mechanotransduction-based substrate from an irreversible scar-based substrate is central to tailoring therapeutic strategies [83].

### 8.4. Practical Management Considerations in AMVP

Risk-adapted management of AMVP should be individualized and primarily driven by arrhythmic burden, myocardial substrate, and valve morphology rather than by the presence of MAD alone [27]. Based on current evidence, the following considerations may guide clinical decision-making:

Medical therapy

- Consider in patients with symptomatic PVCs or non-sustained VAs, suspected PVC-induced cardiomyopathy, reduced LV ejection fraction not explained by MR severity.
- First-line agents include beta-blockers or non-dihydropyridine calcium channel blockers [27].
- Class IC agents or amiodarone may be considered in selected cases, balancing efficacy against long-term adverse effects [27].

Catheter ablation

- May be considered in patients with symptomatic or high-burden PVCs refractory to medical therapy, PVC-induced LV dysfunction, clearly identifiable focal triggers (PMs or Purkinje-related ectopy) [27].
- Its role in preventing SCD remains unproven and should be considered adjunctive rather than definitive therapy.

Implantable cardioverter-defibrillator (ICD)

- Secondary prevention, indicated in patients with prior sudden cardiac arrest, ventricular fibrillation, or sustained VT without reversible causes [27].
- Primary prevention: should be considered on an individualized basis since no specific AMVP phenotype has yet been validated for routine prophylactic ICD implantation.

Mitral valve surgery

- Strongly indicated according to current guidelines in patients with severe degenerative MR with symptoms or LV dysfunction [27].
- May provide arrhythmia-modifying benefit when: VAs are predominantly mechanically driven, there is a marked MAD, annular hypermobility, or PMs traction is present, myocardial fibrosis is absent or limited on CMR [27].

## 9. Unresolved Questions and Future Research Directions

Despite significant advances in the phenotypic characterization of AMVP, several clinically relevant questions remain unanswered and currently limit the development of evidence-based management strategies.

First, the role of prophylactic catheter ablation in patients with high-risk AMVP but without documented sustained VAs remains uncertain. While catheter ablation can effectively suppress PVCs or focal triggers arising from the PMs or Purkinje system, there is no evidence that early ablation alters long-term arrhythmic risk or prevents SCD [83]. Prospective studies are needed to define whether selected phenotypes—such as patients with high ectopic burden, MAD-related mechanical triggers, and absence of established myocardial fibrosis—may benefit from early intervention [84].

Second, the management of athletes with AMVP represents a particularly challenging area. Intense physical activity may exacerbate mechanical stress, adrenergic stimulation, and arrhythmic vulnerability, yet robust data guiding eligibility for competitive sports are lacking [85]. Current recommendations rely largely on extrapolation from general arrhythmia guidelines, underscoring the need for dedicated studies evaluating exercise-induced arrhythmias, dynamic changes in annular–ventricular coupling, and long-term outcomes in this population [85].

Third, the long-term arrhythmic impact of transcatheter mitral valve repair remains poorly defined. While TEER improves survival and symptoms in high-risk patients with degenerative mitral regurgitation, it does not correct MAD or annular–ventricular uncoupling. Whether this translates into persistent or even progressive arrhythmic risk over time requires systematic investigation with extended rhythm surveillance [82].

Fourth, emerging tools such as advanced imaging biomarkers and artificial intelligence–based approaches hold promise for improving risk stratification. Quantitative assessment of myocardial fibrosis, strain abnormalities, inflammatory activity, and annular dynamics—integrated through machine learning models—may allow more precise identification of patients at risk for malignant VAs [26]. However, these strategies remain exploratory and require validation in large, multicenter cohorts before clinical adoption [26].

Finally, there is a critical need for prospective, standardized research frameworks incorporating unified imaging protocols, predefined arrhythmic endpoints, and longitudinal follow-up. Only through such collaborative efforts will it be possible to delineate the natural history of AMVP, identify modifiable risk factors, and define the optimal timing and modality of therapeutic interventions.

## 10. Limitations

This review should be interpreted in light of several limitations. First, although the concept of MVP as a heterogeneous disease spectrum is increasingly supported by contemporary evidence, much of the available data on arrhythmic risk derives from retrospective and observational studies, frequently enriched with high-risk or referral populations [16,17]. This limits the ability to precisely define the prevalence, natural history, and absolute arrhythmic risk across the broader MVP continuum [29]. Second, while MAD emerges as an important phenotypic modifier capable of amplifying mechanical stress and contributing to arrhythmogenic remodelling, its assessment remains affected by substantial heterogeneity in imaging techniques, timing, and quantitative thresholds, preventing its use as a standalone marker of malignant risk [30]. Third, the temporal and causal relationships linking abnormal annular–ventricular coupling, myocardial inflammation, fibrosis development, and ventricular arrhythmias remain incompletely elucidated, as most studies rely on cross-sectional imaging or post-event analyses [64]. Although multimodality imaging has markedly improved phenotypic characterization, prospective validation of individual markers and their integration into standardized risk stratification algorithms is still lacking [65]. Finally, current evidence supporting arrhythmia-modifying interventions—particularly early MV surgery or catheter ablation in the absence of severe regurgitation—is largely indirect and hypothesis-generating, underscoring the need for prospective, multicenter studies with unified imaging protocols, longitudinal follow-up, and predefined arrhythmic endpoints [75].

## 11. Conclusions

MVP should no longer be viewed as a uniform or predominantly benign entity but rather as a heterogeneous disease spectrum in which structural valve abnormalities, annular dynamics, and myocardial remodelling interact to shape clinical outcomes [80]. MAD emerges as an important phenotypic modifier within this continuum, capable of amplifying mechanical stress and promoting arrhythmogenic remodelling, yet insufficient on its own to define malignant risk [81].

The AMVP phenotype arises only in a subset of patients, reflecting the convergence of valve morphology, abnormal annular–ventricular coupling, and myocardial substrate—particularly fibrosis and inflammation. Multimodality imaging plays a pivotal role in disentangling these mechanisms, enabling distinction between potentially reversible, mechanically driven arrhythmias and fixed, scar-based substrates.

Recognizing MVP, MAD, and AMVP as interconnected rather than isolated conditions has important implications for risk stratification and management, supporting a personalized approach that integrates imaging findings, arrhythmic burden, and timing of surgical intervention.

## Figures and Tables

**Table 1:** This table represents the main characteristics of mitral annular disjunction.

MAD	Key Points	References
Definition ^1^	Systolic separation between posterior leaflet hinge point and ventricular myocardium. Mechanical discontinuity. Typically located under posterior scallops, especially P2	[14,16,17,18]
Imaging requirements	Zoomed PLAX or 3-chamber view High frame rate; frame-by-frame analysis Measurement at end-systole Avoid misclassification of redundant leaflet tissue (pseudo-MAD)	[20,24,26]
Factors causing diagnostic variability	Non-uniform definitions across studies Heterogeneity in imaging modalities (TTE vs. cCT vs. CMR). Different disjunction length thresholds Multiplanar modalities systematically detect higher prevalence	[20,21,24]
Pathophysiological implications	Greater longitudinal MAD extent → increased curling, inferolateral systolic outpouching Mechanical stretch Triggering of pro-fibrotic molecular pathways → arrhythmogenic substrate	[10,15,24,25]
Association with fibrosis	Strong correlation between MAD length and CMR fibrosis MAD > 8.5 mm linked to VAs in selected cohort	[10,25]
Prognostic uncertainty	Some studies: MAD associated with malignant VAs Others: no independent association with VAs or SCD MAD may act as marker rather than direct driver	[19,20,21,22,23]

^1^ cCT: cardiac computed tomography, CMR: cardiac magnetic resonance, MAD: mitral annular disjunction, SCD: sudden cardiac death, TTE: trans-thoracic echocardiography, VAs: ventricular arrhythmias.

**Table 2:** This table represents the impact of mitral valve surgery on the arrhythmogenic driver.

Arrhythmogenic Driver	Impact of MV Surgery	Clinical Consequences
MAD/annular–ventricular uncoupling ^1^	Restores annular–LV continuity and corrects paradoxical systolic motion	Reduction in VAs burden when substrate is mechanical and early [74,75,76,77]
Systolic curling of the posterior annulus	Eliminates excessive annular excursion; normalizes hinge-point motion	Decrease in PM traction and mechano-electric triggers [75]
Papillary muscle traction and stretch	Reduction in abnormal traction forces after annuloplasty	Possible suppression of PVC-triggered VAs [76,77]
Dynamic mechanical stretch–driven arrhythmias	Surgical repair removes triggering mechanical forces	VAs reduction observed especially in younger patients (<60 years) [74]
Myocardial inflammation (PET or MRI evidence)	Only partially influenced by surgery; inflammatory substrate persists	Persistent arrhythmias despite mechanically successful repair [78]
Interstitial/replacement fibrosis (LGE elevation)	Unchanged by surgery (fixed electrical substrate)	No significant reduction in arrhythmic risk → ICD/EP strategies needed [74,78]

^1^ ICD: implantable cardioverter device, EP: electrophysiological, LGE: late gadolinium enhancement, MRI: magnetic resonance imaging, MV: mitral valve, PET: positron emission tomography, PM: papillary muscle, PVC: premature ventricular contraction.

## Data Availability

No new data were created or analyzed in this study.

## Abbreviations

3DTEE: three-dimensional transoesophageal echocardiography, AMVP: arrhythmic mitral valve prolapse, cCT: cardiac computed tomography, CMR: cardiac magnetic resonance, ICD: implantable cardioverter defibrillator, EHRA: European Heart Rhythm Association, ILRs: implantable loop recorders, LGE: late gadolinium enhancement, LV: left ventricular, MAD: mitral annular disjunction, MR: mitral regurgitation, MV: mitral valve, MVP: mitral valve prolapse, NYHA: New York Heart Association, NSVT: non sustained ventricular tachycardia, PET-MRI: positron emission tomography-magnetic resonance imaging, PMs: papillary muscles, PVC: premature ventricular contraction, SCD: sudden cardiac death, TEER: transcatheter edge-to-edge repair, TTE: trans-thoracic echocardiography, VAs: ventricular arrhythmias, VF: ventricular fibrillation, VT: ventricular tachycardia.
